# lncRNA MIAT/HMGB1 Axis Is Involved in Cisplatin Resistance *via* Regulating IL6-Mediated Activation of the JAK2/STAT3 Pathway in Nasopharyngeal Carcinoma

**DOI:** 10.3389/fonc.2021.651693

**Published:** 2021-05-20

**Authors:** Xuewei Zhu, Li Liu, Yang Wang, Jianan Cong, Zhang Lin, Yongsen Wang, Qi Liu, Leiming Wang, Ben Yang, Tao Li

**Affiliations:** ^1^ Department of Otolaryngology Head & Neck Surgery, China Japan Union Hospital of Jilin University, Changchun, China; ^2^ Reproductive Medical Center, Department of Gynecology and Obstetrics, China-Japan Union Hospital of Jilin University, Changchun, China; ^3^ Department of Dermatology, The Affiliated Hospital of Changchun University of Chinese Medicine, Changchun, China; ^4^ Department of Ophthalmology, Changchun City Central Hospital, Changchun, China; ^5^ Department of Ophthalmology, China-Japan Union Hospital, Jilin University, Changchun, China; ^6^ Technology Department, Harbin Boshixuan Technology Co., Ltd, Harbin, China; ^7^ Department of Molecular Medicine, University of Texas Health Science Center at San Antonio, San Antonio, TX, United States; ^8^ Shenzhen Bay Laboratory, The Institute of Chemical Biology, Gaoke International Innovation Center, Shenzhen, China; ^9^ Department of Anesthesiology, China-Japan Union Hospital of Jilin University, Changchun, China

**Keywords:** cisplatin resistance, HMGB1, IL6, NPC, lncRNA MIAT

## Abstract

Cisplatin-based chemotherapy and radiotherapy are the main first-line treatment strategies for nasopharyngeal carcinoma (NPC) patients. Unfortunately, resistance is a major obstacle in the clinical management of NPC patients. We prove that the expression level of high-mobility group box 1 (HMGB1) is dramatically increased in resistant NPC cells than that in sensitive cells. HMGB1 induces the expression and secretion of IL6, which leads to constitutive autocrine activation of the JAK2/STAT3 pathway and eventually contributes to chemoresistance in NPC cells. Long non-coding RNAs (lncRNAs) have been identified as key regulators involved in drug resistance. In this study, using GO analysis of the biological process and differential expression analysis, we find 12 significantly altered IncRNAs in NPC cell lines, which may be involved in regulating gene expression. Furthermore, we determine that elevated lncRNA MIAT level upregulates HMGB1 expression, contributing to cisplatin resistance in NPC cells. We find that the deficiency of the lncRNA MIAT/HMGB1 axis, inhibition of JAK2/STAT3, or neutralization of IL6 by antibodies significantly re-sensitizes resistant NPC cells to cisplatin in resistant NPC cells. Moreover, we provide the *in vivo* evidence that the deficiency of HMGB1 reduces cisplatin-resistant tumor growth. Most importantly, we provide clinical evidence showing that the expression level of the lncRNA MIAT/HMGB1/IL6 axis is elevated in resistant NPC tumors, which is highly correlated with poor clinical outcome. Our findings identify a novel chemoresistance mechanism regulated by the lncRNA MIAT/HMGB1/IL6 axis, which indicates the possibilities for lncRNA MIAT, HMGB1, and IL6 as biomarkers for chemoresistance and targets for developing novel strategies to overcome resistance in NPC patients.

## Introduction

Nasopharyngeal carcinoma (NPC) is a type of squamous cell carcinoma that arises from the nasopharynx’s epithelial lining (1). The prevalence of NPC in southern China, Southeast Asia, Northern Africa, Greenland, and Inuits of Alaska endangers human health (1, 2). Clinical evidence and experimental studies indicated that Epstein-Barr virus (EBV) infection, genetic susceptibility, and environmental factors are associated with pathogenic factors of NPC (3, 4). Currently, chemotherapy and radiotherapy are the front-line treatment of NPC. Even though these therapeutic options increased the survival of patients with locally advanced NPC and distant metastasis (DM), resistance to radiotherapy and chemotherapy remains a major clinical obstacle for the treatment of NPC (5–7). Therefore, there is an urgent need to explore the mechanisms underlying radio or chemoresistance for NPC and develop more effective therapeutic strategies.

Recent evidence has revealed that many cytokines/chemokines/growth factors and their receptors are dysregulated in cancers, building up a network involved in regulating tumor progressions, invasion, and especially resistance to radiotherapy and chemotherapy (8, 9). Notably, the interleukin-6 (IL6) cytokine family has been reported to be involved in cancer invasion and proliferation (10). Interestingly, IL6, as a member of the IL6 family and a pleiotropic cytokine, has been shown to accumulate in solid tumors of human colorectal cancer, breast cancer, gastric cancer, prostate cancer, and osteosarcoma (11–14). IL6 is a key player in multiple pathophysiological properties associated with cancer progression and drug resistance (9, 15, 16). The interleukin-6 receptor (IL6R) engages through gp130 ligands and activates the JAK2/STAT3 pathway. IL6R has been shown to be involved in tumor proliferation, survival, invasion, and angiogenesis (17–19). IL6 binding to the IL6R/gp130 complex activates the JAK2/STAT3 pathway and induces its own production in an autocrine manner (13, 20).

High-mobility group box 1(HMGB1) is a nonhistone and highly mobile DNA-binding protein, which functions as a nuclear factor that enhances gene transcription and regulates DNA repair and chromatin remodeling (21, 22). HMGB1 is also recognized as a damage-associated molecular pattern molecule (DAMP) released by necrotic cells and monocytes in response to cell death, inflammation stimuli, and various environmental stressors (21–23). Particularly, dysfunction of intracellular and extracellular HMGB1 has been implicated in the proliferation and metastasis of various cancers, including hepatocellular carcinoma, colorectal cancer, melanoma, and breast cancer (24–27). In many cases, extracellular HMGB1 acts as a pro-tumor protein due to its cytokine, chemokine, and growth factor activity, whereas intracellular HMGB1 promotes drug resistance owing to the pro-autophagy activity and regulation of gene transcription (28–30). However, the function of HMGB1 in chemoresistance remains unknown.

Long non-coding RNAs (lncRNAs), known as non-coding single-strand RNAs, play an essential role as carcinogenic genes or tumor suppressors in the development of human cancer (31–34). Myocardial infarction-associated transcript (MIAT) was first identified as an lncRNA in 2006 and is considered as a regulator in the nucleus interacting with nuclear factors, while as a competitive endogenous RNA (ceRNA) in the cytoplasm (31, 35, 36).

In this study, we demonstrated that the lncRNA MIAT/HMGB1 axis is highly upregulated in cisplatin-resistant nasopharyngeal carcinoma cell lines. The lncRNA MIAT/HMGB1 axis contributes to cisplatin resistance by regulating IL6 expression and secretion. Autocrine IL6 promotes resistance by activating the JAK2/STAT3 pathway in NPC. HMGB1 deficiency overcomes cisplatin resistance *in vivo*. Clinical evidence shows that the expression level of the lncRNA MIAT/HMGB1/IL6 axis is elevated in resistant NPC tumors, which is highly correlated with the poor survival of patients. Thus, the lncRNA MIAT/HMGB1/IL6 axis could be considered as a promising candidate for therapy in chemoresistant NPC.

## Material and Methods

### Cell Cultures

NPC cell lines CNE-2 and HONE-1 were cultured in Roswell Park Memorial Institute 1640 medium with 10% fetal bovine serum (FBS) and 100 IU/ml of penicillin/streptomycin. Cells were maintained at 37°C and 5% CO2 in a humidified incubator. Resistant cells were generated according to previous studies (37). Briefly, CNE-2 resistant cells (CNE-2 CR) and HONE-1 resistant cells (HONE-1 CR) were generated by exposing CNE-2 wild-type cells (CNE-2 WT) or HONE-1 wild-type cells (HONE-1 WT) to increasing concentrations of cisplatin once per week for at least three months.

### Cell Viability Assay

A cell viability assay was performed as previously described (37). Briefly, cells were seeded in a 96-well plate to a final concentration of 5,000 cells per well and cultured to allow adherence. Then cells were exposed to cisplatin at varying concentrations and incubated for 72 h. Cell viability was detected using a Sulforhodamine B (SRB) assay. Cells were fixed by 10% trichloroacetic acid (TCA) then followed by staining using 0.02% SRB, finally the SRB was extracted with Tris buffer (10 mM of tris-HCl, pH 10.5) and the absorbance was measured at 510 nm by a microplate reader (MTX Lab Systems).

### Transfection

Transfections of siRNA were performed with Lipofectamine™ RNAi MAX (Invitrogen) according to the manufacturer’s instructions. Gl2 (luciferase) siRNA was used as a negative control. The HMGB1 plasmid (Addgene #31609) was transfected using Lipofectamine 3000 (Invitrogen) according to the manufacturer’s instructions.

### Generation of HMGB1 Knock-Out Cell Lines

HMGB1 KO cell line CNE-2 was generated using CRISPR/Cas9 as previously described (37).

### qRT-PCR

RNA isolation and qRT-PCR assays were performed as described previously (37). Total RNA from cells was extracted with the miRNeasy Mini Kit (Qiagen), and complementary DNA was synthesized using moloney murine leukemia virus (M-MLV) reverse transcriptase with random primers. cDNA was generated with the BioTeke super RT kit (Bioteke) according to the manufacture’s protocol. qRT-PCR was performed using SYBR Premix Ex TaqTM (TaKaRa). The relative expressions of genes were calculated by normalization to the internal control Actin. Primers are listed in **Supplementary Table 1**.

### Western Blot Analysis

Western blot was performed as described previously (37). The cells were collected in ice-cold PBS, and lysed in RIPA buffer containing protease inhibitor and phosphatase inhibitor cocktail (Santa Cruz Biotechnology). The protein concentrations were determined by use of Bradford methods and boiled in SDS sample buffer (50 mM of Tris [pH 6.8]; 100 mM of DTT; 2% SDS; 0.1% bromophenol blue; 10% glycerol). The proteins were separated on sodium dodecyl sulfate polyacrylamide gel electrophoresis (SDS-PAGE) and transferred onto nitrocellulose membranes. Membranes were then incubated in blocking solution (5% non-fat milk in 20 mM of TRIS-HCl, 150 mM of NaCl, 0.1% Tween-20) (TBST), followed by incubation with the indicated primary antibodies at 4°C overnight. Then secondary antibodies were incubated and detected with an enhanced chemiluminescence reaction.

### Data Mining

The whole data of NPC cell lines CNE-2 and HONE-1 underwent GO analysis of the biological process and differential expression analysis, which were implemented in the R programing language.

### Ethynyl-2’-deoxyuridine (EdU) Cell Proliferation Assay

EdU staining was conducted using the Click-iT™ Plus EdU Alexa Fluor™ 594 Flow Cytometry Assay Kit (Thermo Fisher) as per the manufacturer’s instructions. EdU-positive cells were analyzed by a flow cytometer.

### Xenograft Tumor Model

Eight-week-old nude mice (female, athymic Foxn1nu; Vital River, Beijing, China) were inoculated subcutaneously by injecting CNE-2 CR or CNE-2 CR-KO cells (5 x 10^6^) into the dorsal flank of each mouse. Cisplatin was injected twice per week, for a total of two weeks (5 mg/kg, intraperitoneally) when the tumor volume reached 100 mm^3^. Tumor volume was calculated from caliper measurements by the formula 1/2(L × W2). Tumor volume and body weight were measured twice per week.

### Immunohistochemistry

Immunohistochemistry (IHC) staining was performed as previously described. An antibody against Ki67 was used for specific recognition of corresponding proteins in the xenograft tumor samples. The quantification of cells with positive staining per field was analyzed.

### Nasopharyngeal Carcinoma Patients

The tumor tissues from sensitive and resistant patients were from China Japan Union Hospital of Jilin University. The present study was approved by the institutional ethics committee. Survival rate of patients was analyzed using a Kaplan-Meier plotter (https://kmplot.com/analysis/).

### Statistical Analysis

Data were presented as mean ± SD of at least three independent experiments. Statistical analysis of data was performed by Student’s t test and one-way ANOVA using Graph Pad Prism 7 software for two groups and multiple group comparison. P<0.05 represents the statistically significant difference.

## Results

### Establishment of Cisplatin-Resistant NPC Cells

**Figure 1:**
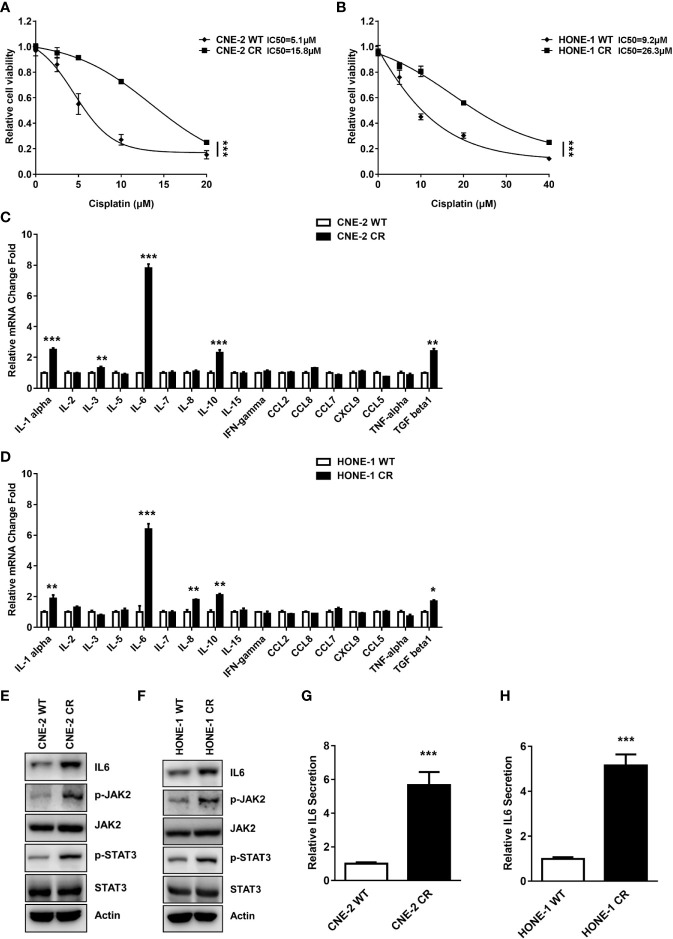
The expression and secretion levels of IL6 were elevated in resistant NPC cells. **(A, B)** Establishment of cisplatin-resistant NPC cells. Cell proliferation was assessed by SRB assay, ***P < 0.001. **(C, D)** The mRNA levels of some cytokines and chemokines in parent wild-type NPC cell lines (CNE-2 WT and HONE-1 WT) and resistant cell lines (CNE-2 CR and HONE-1 CR) were detected by qPCR, *P < 0.05, **P < 0.01, ***P < 0.001. **(E, F)** The protein levels of IL6, p-JAK2, JAK2, p-STAT3, STAT3, and Actin were detected by western blotting. **(G, H)** IL6 secretion levels were detected by an ELISA kit, ***P < 0.001.

To discover new mechanisms of resistance, pathways, and targets for novel drug combinations that can overcome cisplatin resistance, we established resistant NPC cells using two parent NPC cell lines CNE-2 and HONE-1, according to methods described previously (37). We obtained sub-cell lines, CNE-2 CR and HONE-1 CR, which could grow in a high concentration of cisplatin (**Figures 1A, B**). The SRB cell survival assay revealed that the IC50 of cisplatin in CNE-2 CR and HONE-1 CR increased 3-fold (**Figures 1A, B**). We further used these two pairs of cell lines to screen for factors that may contribute to chemoresistance.

### Identification of IL6 as a Key Factor That May Regulate Cisplatin Resistance

Cytokines and chemokines have been reported to participate in the progression of tumor growth, invasion, metastasis, and drug resistance. The expressions of cytokines and chemokines including IL6, IL-11, TGF-b, IL-5, IL-10, and IL-1b have significant differences after ionizing radiation (IR) or cisplatin treatment in various tumors (8, 11, 38, 39). Thus, cytokines or chemokines are designated as potential biomarkers for diagnosis and treatment in cancers. We hypothesized that cytokines or chemokines and their activated receptors might be involved in resistance to cisplatin. To further test this possibility, we detected the mRNA expression levels of some cytokines and chemokines in parent wild-type sensitive NPC cell lines (CNE-2 WT and HONE-1 WT) and resistant cell lines (CNE-2 CR and HONE-1 CR). Interestingly, the IL6 mRNA level was significantly increased in both resistant cell lines compared to the sensitive cell lines (**Figures 1C, D**), indicating that an elevated level of IL6 may cause resistance.

### The Expression and Secretion Levels of IL6 Are Elevated in Resistant NPC Cells

To verify if IL6 expression and its downstream pathway are upregulated in cisplatin resistant cells, we detected the IL6 protein level and its downstream pathway in cisplatin sensitive and resistant cells. Consistently, we found the protein level of IL6 was significantly increased in both resistant cell lines (**Figures 1E, F**). Furthermore, we tested the activation of IL6 downstream signaling *via* the JAK2/STAT3 pathway. The phosphorylation levels of JAK2 (Y1007/1008) and STAT3 (Y694) were both upregulated in resistant cells, while the total protein level of JAK2 and STAT3 were not altered when compared to the sensitive cells (**Figures 1E, F**). Our data indicated that IL6 is upregulated, which activates the JAK2/STAT3 pathway in resistant cells.

Given that IL6 mRNA and protein levels are increased in both resistant cells, we hypothesized that IL6 in the medium of resistant cells may cause, in an autocrine fashion, the activation of a downstream pathway, which eventually results in resistance to cisplatin. We tested the level of IL6 secreted in resistant cells. Consistently, the secretion level of IL6 in the medium of both resistant cells was significantly elevated compared to their sensitive counterparts (**Figures 1G, H**).

### Autocrine IL6 Activation Promotes Cisplatin Resistance

**Figure 2:**
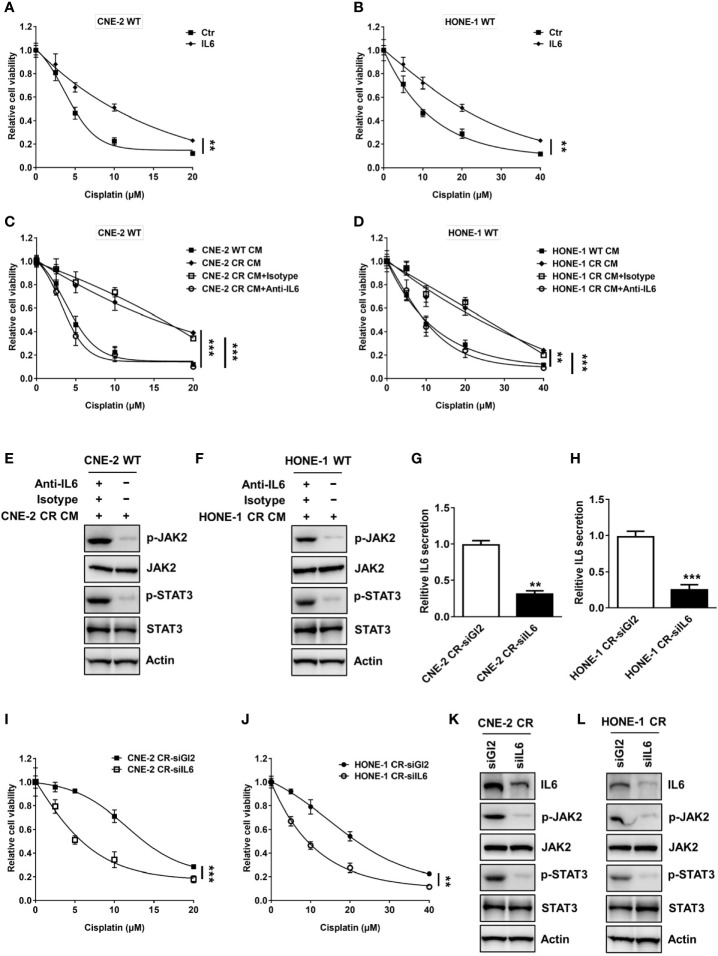
Activation of IL6 promotes cisplatin resistance. **(A, B)** Cell proliferation was assessed by SRB assay after recombinant IL6 treatment, **P < 0.01. **(C, D)** Cell proliferation was assessed by SRB assay after incubation with conditional medium from wild-type cells or resistant cells, and isotype control or anti-IL6, **P < 0.01, ***P < 0.001. **(E, F)** The protein levels of p-JAK2, JAK2, p-STAT3, STAT3, and Actin were detected by western blotting in CNE-2 WT and HONE-1 WT cells treated with conditional medium from resistant cells, and isotype control or anti-IL6. **(G, H)** IL6 secretion levels were detected by an ELISA kit after IL6 siRNA transfection, ***P < 0.001. **(I, J)** Cell proliferation was assessed by SRB assay after IL6 siRNA transfection, ***P < 0.001. **(K, L)** The protein levels of IL6, p-JAK2, JAK2, p-STAT3, STAT3, and Actin were detected by western blotting after IL6 siRNA transfection.

Given the above findings of the elevated expression and secretion levels of IL6, we hypothesized that IL6-mediated autocrine activation of the JAK2/STAT3 pathway may contribute to the induction of resistance in NPC. We showed that the stimulation of recombinant IL6 treatment turned the sensitive cells toward resistance to cisplatin (**Figures 2A, B**). To confirm whether the elevated IL6 secretion level in the medium of resistant cells causes resistance to cisplatin, the conditioned medium from sensitive cells and resistant cells were collected to culture sensitive cells. Indeed, both sensitive cells CNE-2 WT and HONE-1 WT cultured with the conditioned medium from resistant cells displayed an elevated resistance to cisplatin, compared to the cells cultured in the conditioned medium from sensitive cells (**Figures 2C, D**). However, this elevated resistance was abolished by an IL6 neutralization antibody (**Figures 2C, D**). Meanwhile, the activation level of the JAK2/STAT3 pathway was also dramatically decreased by an IL6 neutralization antibody (**Figures 2E, F**). These results indicate that the secreted IL6 acts as an autocrine factor to induce cisplatin resistance in NPC cells *via* the JAK2/STAT3 pathway. Furthermore, we found that IL6 deficiency by specific siRNA resulted in a decreased IL6 secretion level (**Figures 2G, H**). More importantly, IL6 deficiency suppressed the cell survival of both resistant cell lines by downregulating the activation of the JAK2/STAT3 pathway, indicating that IL6 deficiency re-sensitizes resistant cells to cisplatin (**Figures 2I–L**).

### HMGB1 Upregulates the Expression of IL6 in NPC Resistance

**Figure 3:**
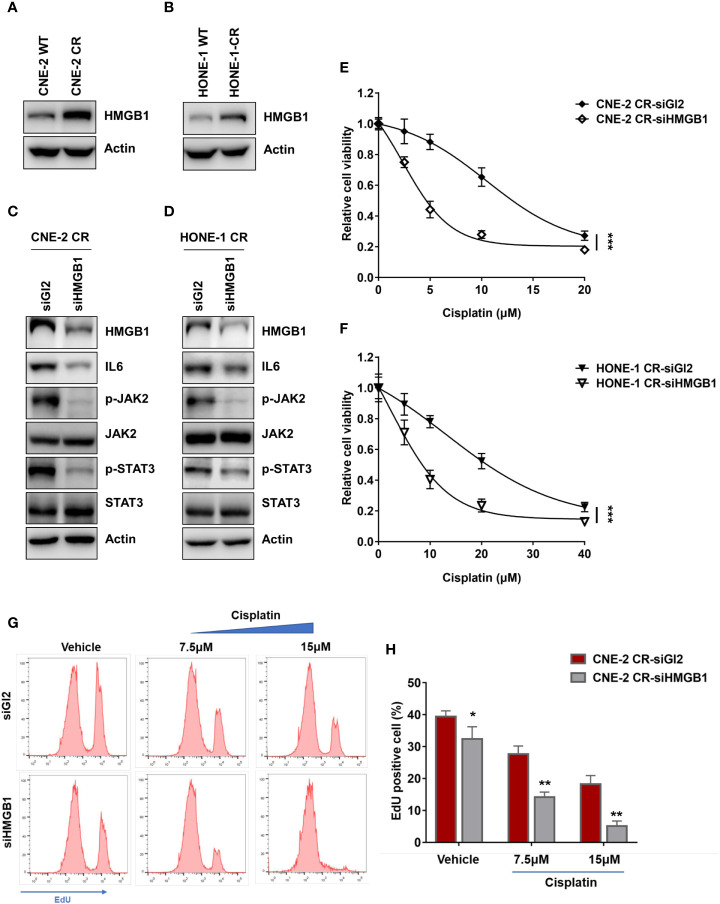
HMGB1 is upregulated in NPC cisplatin-resistant cells. **(A, B)** The protein levels of HMGB1 and Actin were detected by western blotting. **(C, D)** The protein levels of HMGB1, IL6, p-JAK2, JAK2, p-STAT3, STAT3, and Actin were detected by western blotting after HMGB1 siRNA transfection. **(E, F)** Cell proliferation was assessed by SRB assay, after HMGB1 siRNA transfection ***P < 0.001. **(G, H)** A cell proliferation assay was performed using an EdU assay and analyzed by flow cytometry after siRNA transfection. *P < 0.05, **P < 0.01.

**Figure 4:**
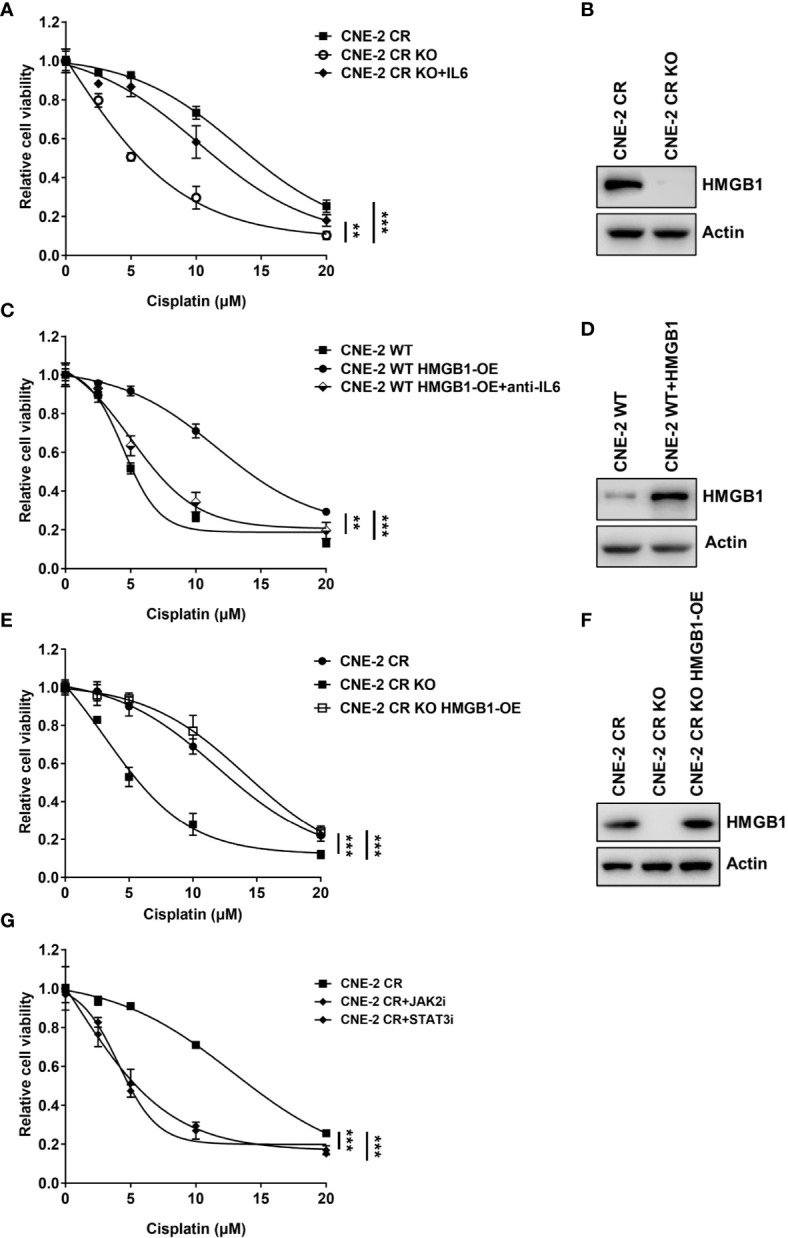
HMGB1 upregulates expression of IL6 in resistance. **(A)** Cell proliferation was assessed by SRB assay after recombinant IL6 treatment in CNE-2 CR and CNE-2 CR-KO cells, **P < 0.01, ***P < 0.001. **(B)** The protein levels of HMGB1 and Actin were detected by western blotting in CNE-2 CR and CNE-2 CR-KO cells. **(C)** Cell proliferation was assessed by SRB assay after anti-IL6 treatment in CNE-2 WT and CNE-2 HMGB1 OE cells, **P < 0.01, ***P < 0.001. **(D)** The protein levels of HMGB1 and Actin were detected by western blotting after HMGB1 was overexpressed in CNE-2 WT cells. **(E)** Cell proliferation was assessed by SRB assay after HMGB1 was overexpressed in CNE-2 CR OK cells. **(F)** The protein levels of HMGB1 and Actin were detected by western blotting in CNE-2 CR and CNE-2 CR KO and CNE-2 CR KO with HMGB1 overexpressed cells. **(G)** Cell proliferation was assessed by SRB assay after treatment with JAK2 inhibitor LY2784544 or STAT3 inhibitor stattic, ***P < 0.001.

Previous studies have reported that HMGB1 is associated with cytokines or chemokines production (21, 23, 28, 40–42). We tested whether HMGB1 is involved in the upregulation of IL6. Interestingly, we observed that the basal levels of HMGB1 in both resistant cells CNE-2 CR and HONE-1 CR were significantly elevated (**Figures 3A, B**). To determine whether HMGB1 regulates IL6 expression, we knocked down HMGB1 with siRNA and detected the expression of IL6 and the activation of the downstream JAK2/STAT3 pathway. HMGB1 deficiency significantly reduced IL6 expression, as well as the phosphorylation level of JAK2 and STAT3 (**Figures 3C, D**). Importantly, HMGB1 deficiency re-sensitized resistant cells to cisplatin (**Figures 3E, F**). Similar results were observed from the EdU cell proliferation assay. The resistant cells with HMGB1 deficiency showed less proliferation when exposed to cisplatin compared to the cells in the control group (**Figures 3G, H**). To confirm whether HMGB1 contributes to cisplatin resistance in NPC cells *via* targeting IL6 directly, we established HMGB1 knock-out cells in resistant NPC cells (CNE-2 CR KO) for rescue experiments (**Figures 4A, B**). The results showed that HMGB1 KO cells in resistant cells re-sensitized to cisplatin, which was polished by recombinant IL6 treatment. Consistently, the elevated resistance caused by ectopic overexpression of HMGB1 in sensitive CNE-2 WT cells was polished by the treatment of the IL6 neutralization antibody (**Figures 4C, D**). To further validate the HMGB1 function, we rescued HMGB1 by overexpressing it in HMGB1 KO resistant cells. Results showed that overexpressed HMGB1 impaired the sensitivity to cisplatin in HMGB1 KO resistant cells (**Figures 4E, F**). Taken together, these results suggest that HMGB1 contributes to cisplatin resistance by directly upregulating IL6 expression in NPC cells.

Given that HMGB1 elevated IL6 level, which induces the activation of the JAK2/STAT3 pathway, we explored the approach to overcome cisplatin resistance *via* treating resistant cells with JAK2 inhibitor LY2784544 or STAT3 inhibitor stattic. Similarly, both the JAK2 inhibitor and STAT3 inhibitor exhibited a synergistic effect with cisplatin on resistant cells (**Figure 4G**) which suggests possibilities to develop novel drug combinations with chemotherapy to overcome resistance in NPC.

### HMGB1 Deficiency Overcomes Cisplatin Resistance *In Vivo*

**Figure 5:**
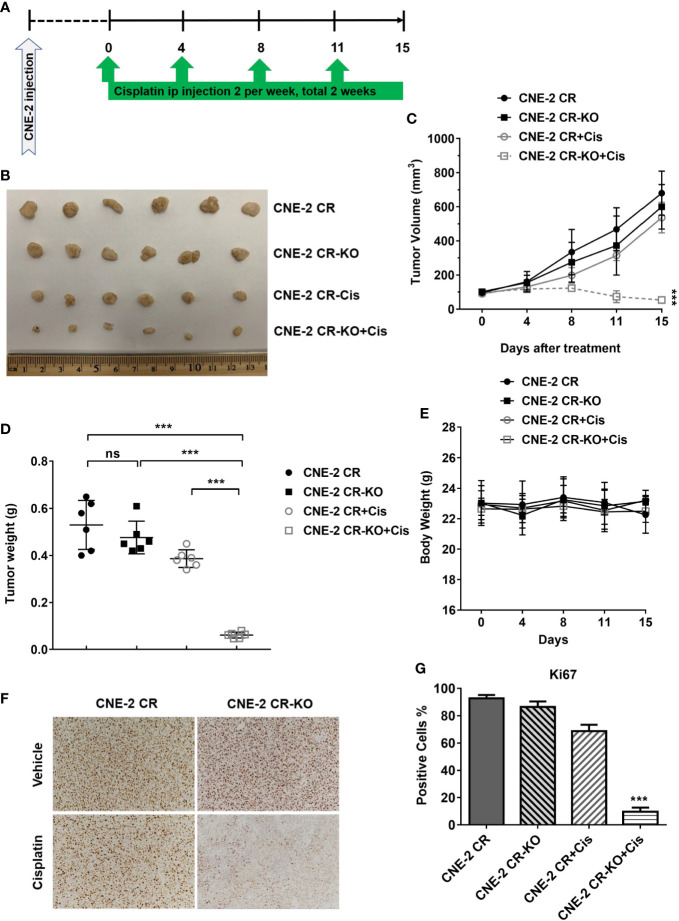
HMGB1 deficiency overcomes cisplatin resistance *in vivo*. **(A)** The scheme of xenograft tumors and treatment. **(B)** The photograph of the representative tumors from mice in each treatment group. Ruler scale is shown in cm. **(C)** Growth curves of CNE-2 CR xenograft tumors treated with vehicle and cisplatin (5 mg/kg, intraperitoneally) for two weeks, n = 6 mice/group, ***P < 0.001. **(D)** The tumor weight in each treatment group, ***P < 0.001. **(E)** The body weight from mice in each treatment group. **(F–G)** Immunohistochemistry staining of Ki67 in xenograft tumor samples. Ki67 positive cells were quantified. NS, Non-significant.

To evaluate whether HMGB1 deficiency could re-sensitize cisplatin *in vivo*, we established xenograft tumors by injecting CNE-2 CR or CNE-2 CR-KO cells, followed by cisplatin treatment (**Figure 5A**). We found that HMGB1 deficiency without cisplatin treatment did not affect tumor growth. Strikingly, HMGB1 deficiency showed a dramatically synergistic effect with cisplatin in resistant xenograft tumors with no significant difference in body weight (**Figures 5B–E**). Similarly, the Ki67 immunohistochemistry (IHC) results showed that cell proliferation was impaired in CNE-2-KO xenograft tumors with cisplatin treatment (**Figures 5F, G**).

### lncRNAs Differentially Express in NPC Cells

**Figure 6:**
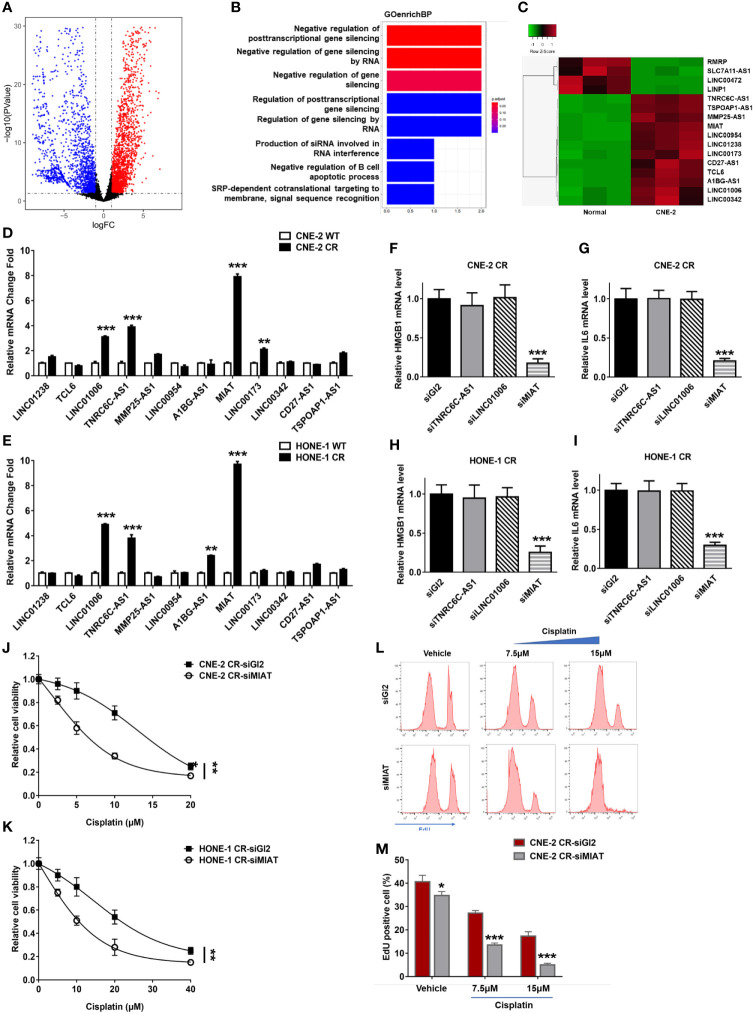
lnc MIAT contributes to cisplatin resistance *via* regulating HMGB1 in NPC cells. **(A)** Volcano plot showing differentially expressed lncRNAs in CNE-2 compared with normal nasopharynx cells. A change is considered significant if the change is >2-fold with a p-value of <0.05. **(B)** GO analysis of the biological process in CNE-2 cells. **(C)** Heatmap showing the selected differentially expressed lncRNAs in CNE-2 after overlap with those in HONE-1. **(D, E)** lncRNA levels were detected by qPCR, **P < 0.01, ***P < 0.001. **(F–I)** The expression levels of HMGB1 and IL6 were detected by qPCR after siRNA transfection of lncRNAs, **P < 0.01, ***P < 0.001. **(J, K)** Cell proliferation was assessed by SRB assay, after MIAT siRNA transfection ***P < 0.001. **(L, M)** Cell proliferation assay was performed using an EdU assay and analyzed by flow cytometry after siRNA transfection. *P < 0.05.

lncRNAs have been reported to regulate the growth, proliferation, invasion, metastasis, and drug resistance of various cancers (32, 33, 34). lncRNAs participate in transcriptional modulation, splicing regulation, post-transcriptional process, chromatin remodeling, or protein-protein, protein-DNA, and protein-RNA interactions (43, 44, 45). We, therefore, compared the nasopharyngeal carcinoma cell lines CNE-2 and HONE1 to a normal nasopharynx cell line for the detection of differentially expressed lncRNAs (**Figure 6A** and **Supplementary Figure 1**). The GO analysis results showed differentially expressed genes enrichment in the pathway of negative regulation of posttranscriptional gene silencing and negative regulation of gene silencing by RNA, which had been reported to be associated with chemotherapy resistance in various cancers (**Figure 6B**). This result indicates that these lncRNAs may be involved in the regulation of HMGB1 expression. We overlapped the differentially expressed lncRNAs in CNE-2 and HONE-1 cell lines. Twelve lncRNAs (LINC01238, TCL6, LINC01006, TNRC6C-AS1, MMP25-AS1, LINC00954, A1BG-AS1, lnc MIAT, LINC00173, LINC00342, CD27-AS1, and TSPOAP1-AS1) were found dramatically differentially expressed compared with the normal nasopharynx cell line, which may be involved in the regulation of HMGB1 expression (**Figure 6C**).

### lncRNA MIAT Contributes to Cisplatin Resistance *via* Regulating HMGB1 in NPC Cells

To determine the roles of lncRNAs involved in cisplatin resistance in NPC cells, we tested the expression levels of these twelve lncRNAs in resistant NPC cells. We found that TNRC6C-AS1, LINC01006, and lncRNA MIAT were upregulated in both resistant NPC cells CNE-2 CR and HONE-1 CR (**Figures 6D, E**). To determine which lncRNA may be involved in regulating HMGB1 in resistant cells, we detected their effect on HMGB1 expression *via* defecting these lncRNAs by siRNA individually. Notably, lncRNA MIAT deficiency decreased HMGB1 expression as well as IL6 expression, while TNRC6C-AS1 and LINC01006 did not show an effect on HMGB1 and IL6 (**Figures 6F–I**). This result indicates that lncRNA MIAT participates in HMGB1 expression in resistance. To further confirm whether lncRNA MIAT deficiency overcomes resistance, we tested synergy with cisplatin in NPC resistant cells and results showed that lncRNA MIAT deficiency re-sensitized cells to cisplatin (**Figures 6J, K**). Similar results were obtained from the EdU cell proliferation assay. The resistant cells with lncRNA MIAT deficiency showed less proliferation when exposed to cisplatin (**Figures 6L, M**). Taken together, we found that lncRNA MIAT participated in cisplatin resistance *via* regulating the HMGB1/IL6 axis in NPC cells.

### Clinical Evidence of the lncRNA MIAT/HMGB1/IL6 Axis

**Figure 7:**
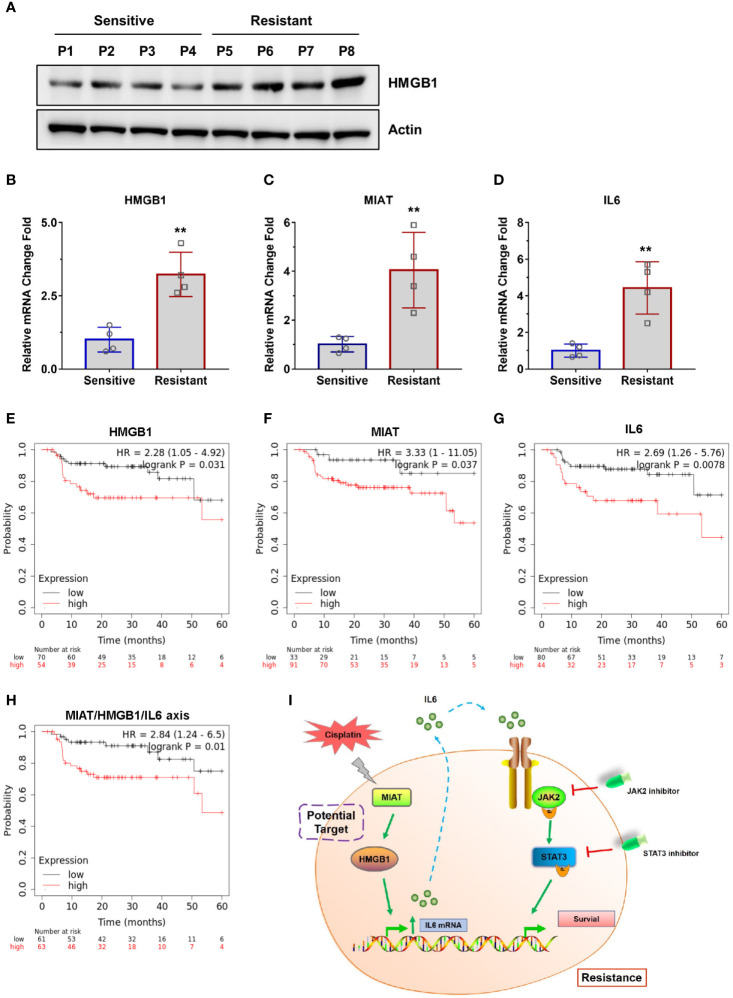
Clinical evidence of the lncRNA MIAT/HMGB1/IL6 axis. **(A)** The protein levels of HMGB1 and Actin were detected by western blotting in patient samples. **(B–D)** The mRNA levels of IL6, HMGB1, and lncRNA MIAT were detected by qPCR in patient samples, **P < 0.01. **(E–H)** Survival rate of patients was analyzed using a Kaplan-Meier plotter (https://kmplot.com/analysis/). **(I)** Working model of the lncRNA MIAT/HMGB1/IL6 axis in regulating cisplatin resistance in NPC cells.

To directly evaluate the correlation between the lncRNA MIAT/HMGB1/IL6 axis and tumor resistance in NPC patients, we collected samples from cisplatin-sensitive patients and the patients developed resistance. We obtained four sensitive tumor samples and four resistant tumor samples and analyzed the expression levels of lncRNA MIAT, HMGB1, and IL6 (**Figures 7A–D**). We found that all the resistant patients had elevated lncRNA MIAT, HMGB1, and IL6 expression levels. Inspired by this finding of the correlation between the lncRNA MIAT/HMGB1/IL6 axis and resistance in NPC patients, we further evaluated the correlation between the expression of the lncRNA MIAT/HMGB1/IL6 axis and survival rate. NPC is the most common head neck squamous cell carcinoma (HNSCC) derived from the epithelial cells at the nasopharynx. Given that NPC samples are relatively rare to obtain, we analyzed the data from HNSCC patients in the datasets and correlated the expression levels of lncRNA MIAT, HMGB1, and IL6 with the survival rate of these patients using a Kaplan-Meier plotter (https://kmplot.com/analysis/). Indeed, patients exhibited a worse 5-year PPS rate when the expression level of lncRNA MIAT, HMGB1, or IL6 was higher (**Figures 7E–G**). Consistently, the high expression level of the lncRNA MIAT/HMGB1/IL6 axis significantly correlated with poor patient survival (**Figure 7H**). Thus, this clinical evidence strongly indicates that the expression level of the lncRNA MIAT/HMGB1/IL6 axis is elevated in resistant NPC tumors, which is highly correlated with poor survival.

## Discussion

In this study, we showed the elevated lncRNA MIAT/HMGB1 axis is responsible for IL6-mediated activation of the JAK2/STAT3 pathway to endow NPC cells with cisplatin resistance (**Figure 7I**). We have provided the evidence that targeting lncRNA MIAT, HMGB1, or IL6 is capable of re-sensitizing cells to cisplatin. In recent years, there has been more and more evidence that lncRNAs can be considered as new and valuable molecules that are involved in the regulation of the growth, proliferation, invasion, metastasis, and drug resistance of cancer cells (32, 33, 34). lncRNAs participate in transcriptional modulation, splicing regulation, post-transcriptional process, chromatin remodeling, or protein-protein, protein-DNA, and protein-RNA interactions (43, 44, 45). Myocardial infarction-associated transcript (MIAT) known as retina non-coding RNA2, locates on the human chromosome 12q12.1 (31, 46). It has been reported that lncRNA MIAT acts as an oncogene upregulated in various cancers such as gastric cancer, liver cancer, colorectal cancer, and lung cancer (31). lncRNA MIAT is involved in regulating proliferation, migration, invasion, drug resistance, and cell cycle by targeting some proteins, including MMP9, SF1, TDP43, ZEB1, MYO1B, SGK1, WNT9A, DDX5, and HDAC4 (31). However, the role of lncRNA MIAT in cisplatin-resistant NPC cells is barely known. For the first time, our study determined that MIAT, as well as HMGB1, is upregulated in cisplatin-resistant NPC cells. More importantly, we have revealed that MIAT contributes to cisplatin resistance *via* upregulating HMGB1 expression and then elevating IL6 expression and secretion levels. However, the mechanism by which MIAT regulates HMGB1 expression remains to be defined in a future study. Given that MIAT has been revealed to play a regulatory role in the cytoplasm *via* the mechanism of competitive endogenous RNA (ceRNA) to target various microRNAs including miR-141, miR-29A-3p, miR-133a-5p, miR-184, miR-34a, miR-1246, and miR-150 (31), we assume that MIAT regulates HMGB1 expression *via* targeting some microRNAs.

HMGB1 was first discovered as one of a group of chromatin-associated proteins. Structurally, HMGB1 protein contains two homologous DNA-binding domains with a negatively charged C-terminal region (47). Nuclear HMGB1 binds to DNA to stabilize the nucleosome and regulate chromatin remodeling, DNA repair, and gene transcription (22). HMGB1 has been shown to be a transcriptional co-factor of multiple genes, including p53, p73, NF-κB, and estrogen receptor (22, 30). Therefore, it is reasonable that HMGB1 participates in upregulating IL6 expression. Most importantly, for the first time, we demonstrated that the expression level of HMGB1 is much higher in resistant NPC cells than in sensitive cells, suggesting that HMGB1 may be associated with resistance. Indeed, we have found that HMGB1 deficiency re-sensitizes resistant cells to cisplatin. Extracellular HMGB1 also acts as a pro-tumor protein due to its cytokine, chemokine, and growth factor activity (28–30). It has been previously reported that HMGB1 binds to lipoteichoic acid and enhances TNF-α and IL6 production in an inflammatory response (40). Consistent with this report, our findings have revealed that HMGB1 upregulates IL6 expression to contribute to resistance, because HMGB1 has dual nucleus and extracellular functions. This also opens up new possibilities that the secreted HMGB1 may also improve tumor cell survival and invasion. Moreover, we found that HMGB1 deficiency overcomes cisplatin resistance *in vivo*, which provides evidence that HMGB1 could be a potential target for the following therapy of NPC patients.

We have also provided evidence that IL6 acts as an autocrine factor to activate the downstream JAK2/STAT3 pathway, which eventually transforms sensitive cells into resistant cells. Therefore, it provides the possibility that IL6 can be potentially used as a biomarker for diagnosing, preventing, and treating cisplatin resistance in clinical application.

Most importantly, we have provided clinical evidence showing that the expression level of the lncRNA MIAT/HMGB1/IL6 axis is elevated in resistant NPC tumors, which is highly correlated with the poor survival of patients. Therefore, lncRNA MIAT, HMGB1, and IL6 are promising targets in drug development to overcome resistance in NPC patients. Additionally, it is well known that the JAK inhibitor (LY2784544) we used in this study is currently in a Phase 2 clinical trial for its potential use in treating myeloproliferative neoplasms (NCT01594723). During the Phase 1 trials, patients showed good tolerance to LY2784544 and clinical improvement at a dose of 120 mg/day (48). Our studies have indicated that LY2784544 can efficiently re-sensitize cisplatin-resistant NPC cells to cisplatin *via* inhibition of JAK2, which eventually blocks IL6 autocrine activity. Therefore, the application of specific JAK2 inhibitors, including LY2784544 is likely to lead to a promising and efficient combination approach with chemotherapy as the first-line treatment or the treatment strategy for relapsed patients after radiotherapy (**Figure 7I**). We have also demonstrated that the STAT3 inhibitor (stattic) overcomes resistance, which indicates that the STAT3 inhibitor is promising for drug innovation (**Figure 7I**). Taken together, our study provides the impetus to discover new strategies, for instance, the development of efficient agents, including JAK2/STAT3 inhibitors and humanized monoclonal antibodies against IL6 to re-sensitize resistant NPC cells or other cancers that were resistant to chemotherapy.

## Data Availability Statement

The original contributions presented in the study are included in the article/**Supplementary Material**. Further inquiries can be directed to the corresponding authors.

## Ethics Statement

The animal study was reviewed and approved by Laboratory Animal Welfare Ethics Committee of Jilin University. The patients study was approved by the institutional ethics committee of Jilin University.

## Author Contributions

BY and TL designed the project. XZ, LL, YW, JC, ZL, YSW, QL, and LW performed the experiments and analyzed the data. XZ and LL prepared the manuscript. XZ and YSW performed the experiments in manuscript revision process. BY and TL contributed to manuscript revision. All authors contributed to the article and approved the submitted version.

## Conflict of Interest

Author YSW was employed by the company Harbin Boshixuan Technology Co.

The remaining authors declare that the research was conducted in the absence of any commercial or financial relationships that could be construed as a potential conflict of interest
