# Supplementary material for: lncRNA MIAT/HMGB1 Axis Is Involved in Cisplatin Resistance via Regulating IL6-Mediated Activation of the JAK2/STAT3 Pathway in Nasopharyngeal Carcinoma
Source: Front Oncol. 2021 May 20;11:651693. doi: 10.3389/fonc.2021.651693 (PMC8173225; doi:10.3389/fonc.2021.651693)
Supplement: Supplementary file 1 [file DataSheet_1.docx]

**Supplementary materials**

**
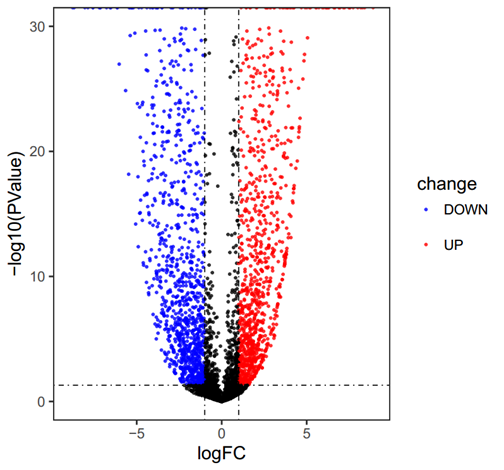
**

**Supplemental figure legends**

**Figure S1.** Volcano plot showing differentially expressed lncRNAs in HONE-1 compared with normal nasopharynx cells. A change is considered significantly if the change is >2-fold with a p-value <0.05.


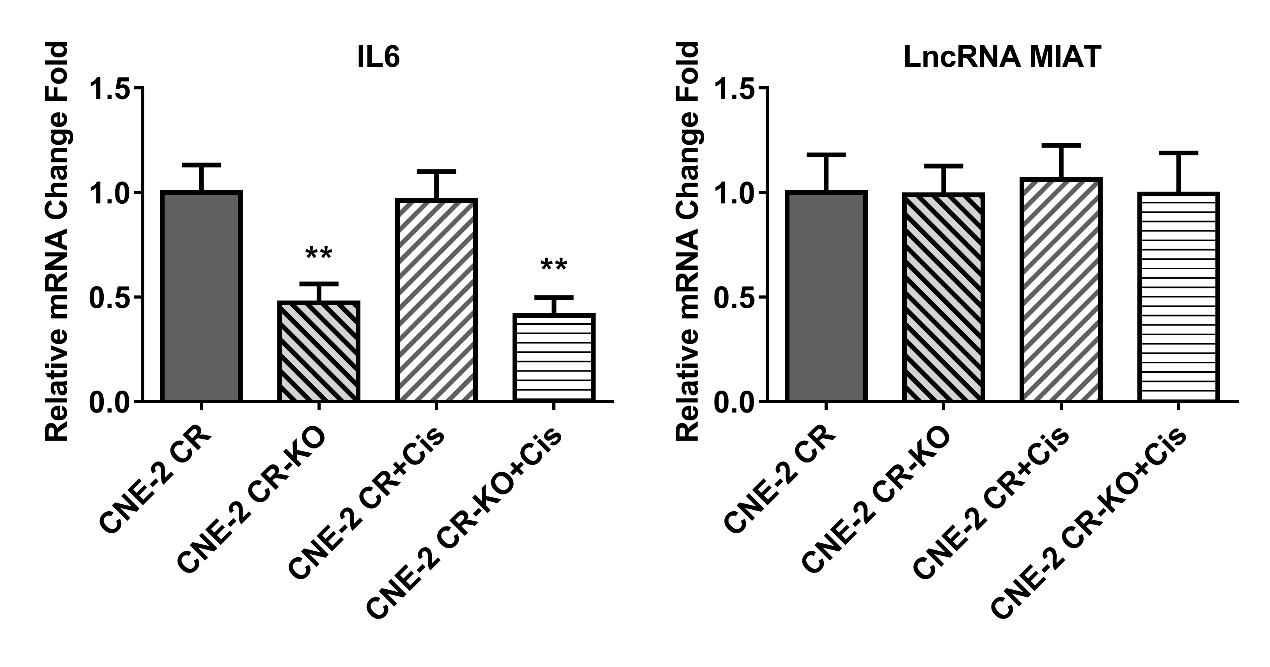


**Figure S2.** The expression levels of IL6 and LncRNA MIAT in xenograft tumors were detected by qPCR, ** P ˂ 0.01.

| qRT-PCR primer sequences (all sequences from 5′to 3′) | | |
| --- | --- | --- |
| RT-PCR Primers | Forward | Reverse |
| IL-1 alpha | GACGCACTTGTAGCCACGTA | GGCCATCTTGACTTCTTTGCT |
| IL-2 | CCAAGAAGGCCACAGAACTGA | TTGGGAAGAAGGTTTGTGTTCC |
| IL-3 | CACCCACGCGACATCCAAT | AGAGAACGAGCTGGACGTTG |
| IL-5 | TACGTGTATGCCATCCCCAC | TCCTCAGAGTCTCATTGGCT |
| IL-6 | GGAGAGGGAGCGATAAACACA | CTCAGACATCTCCAGTCCTCT |
| IL-7 | GCATCGATCAATTATTGGACAGC | TGCGAGCAGCACGGAATAA |
| IL-8 | TCAGAGACAGCAGAGCACAC | CTTGGCAAAACTGCACCTTCA |
| IL-10 | TACGGCGCTGTCATCGATTT | ACTCATGGCTTTGTAGATGCCT |
| IL-15 | GCCAGGACTCGATGGAGAATC | CGATCTTGTATGGGCTGGCT |
| IFN-γ | GGCTTTTCAGCTCTGCATCG | TCTGTCACTCTCCTCTTTCCAA |
| CCL2 | TCTCAAACTGAAGCTCGCACT | TGGGGCATTGATTGCATCTGG |
| CCL8 | CCCAAGGAAGCTGTGATCTTCA | TGGAATCCCTGACCCATCTCT |
| CCL7 | TCTCATGTGGAAGCCCATGC | GTATTAATCCCAACTGGCTGAGCA |
| CXCL9 | AGTGCAAGGAACCCCAGTAG | AGGGCTTGGGGCAAATTGTT |
| CCL5 | CAGTCGTCCACAGGTCAAGG | CTTGTTCAGCCGGGAGTCAT |
| TGFβ | GGAAATTGAGGGCTTTCGCC | CCGGTAGTGAACCCGTTGAT |
| TNFα | CCCATGTTGTAGCAAACCCT | TTATCTCTCAGCTCCACGCC |
| LINC01238 | TAACCTCAAAGCAGAGGCCG | CTGGGCAGCACTGAACTACA |
| TCL6 | TGTCTCATTCGCCTCTGGAT | GTCTCCCTCCTTCTGCCTTT |
| LINC01006 | GTCGAGGGCAGTTTTCTGGG | AACACAACCCGCAACGAGAA |
| TNRC6C-AS1 | GGGTCTAGCCCACCCAATC | ATGGGCTCAACAGGTCACAA |
| MMP25-AS1 | GAGGCCTGAAGAGACCAAGAC | AGTGCACCGGAAGTTACAGA |
| LINC00954 | CATGATGGCCACAGACCCTT | GCTGGTGACGTCAAGTCAGA |
| A1BG-AS1 | CATCCTCTGCCCAAACTCCTT | GGAGGATACAGGGGGCATGA |
| MIAT | GAGGGAAGTTCTGAGCTTGG | CCTTTCTTCTGGGCTGAGAC |
| LINC00173 | GCCAGCTCTCGGTACCTGGA | GGATCGCAACATTCCTGCCAAG |
| LINC00342 | CCCAAAGCAGTCCTTCACTACA | GCAGTTCACTCTGCTGCTT |
| CD27-AS1 | GGACCCCATAGGGCACATCT | GTACTCCCCACTCCTACCCC |
| TSPOAP1-AS1 | TGTCTGCATCCCACAACAGG | CAAGCAGATTGCTGCCTTGC |
| Actin | CACCATTGGCAATGAGCGGTTC | AGGTCTTTGCGGATGTCCACGT |
| U6 | GCTTCGGCAGCACATATACTAAAAT | CGCTTCACGAATTTGCGTGTCAT |

**Supplemental table.**

**qRT-PCR primer sequences.**
